# Severely disabling chronic pain in young adults: prevalence from a population-based postal survey in North Staffordshire

**DOI:** 10.1186/1471-2474-6-42

**Published:** 2005-07-21

**Authors:** Christian Mallen, George Peat, Elaine Thomas, Peter Croft

**Affiliations:** 1 Primary Care Sciences Research Centre, Keele University, Staffordshire, UK

## Abstract

**Background:**

Severely disabling chronic pain in the adult population is strongly associated with a range of negative health consequences for individuals and high health care costs, yet its prevalence in young adults is less clear.

**Methods:**

All adults aged 18–25 years old registered with three general practices in North Staffordshire were invited to complete a postal questionnaire containing questions on pain within the last 6 months, pain location and duration. Severity of chronic pain was assessed by the Chronic Pain Grade. Severely disabling chronic pain was defined as pain within the last six months that had lasted for three months or more and was highly disabling-severely limiting (Grade IV).

**Results:**

858 responses from 2,389 were received (adjusted response = 37.0%). The prevalence of any pain within the previous six months was 66.9% (95%CI: 63.7%, 70.1%). Chronic pain was reported by 14.3% (95%CI: 12.0%, 16.8%) of respondents with severely disabling chronic pain affecting 3.0% (95%CI: 2.0%, 4.4%) of this population. Late responders were very similar to early responders in their prevalence of pain. Cross-checking the practice register against the electoral roll suggested register inaccuracies contributed to non-response.

**Conclusion:**

Pain is a common phenomenon encountered by young adults, affecting 66.9% of this study population. Previously observed age-related trends in severely disabling chronic pain in older adults extend to younger adults. Although a small minority of younger adults are affected, they are likely to represent a group with particularly high health care needs. High levels of non-response in the present study means that these estimates should be interpreted cautiously although there was no evidence of non-response bias.

## Background

Chronic pain has been estimated to affect between 2–40% of the adult population [1]. Although different definitions of chronic pain have been used in these epidemiological studies, differentiating chronic pain on the basis of global severity appears to identify important subgroups [2]. Compared with chronic pain of mild intensity and minimal disability, individuals with severely disabling chronic pain are more likely to have long-term limiting illness and comorbid health conditions, to report poorer self-rated health, mental well-being and social functioning, have higher levels of depression and work loss, and account for a large proportion of total and condition-specific health care costs and utilisation [3-9].

Young adults are an important group to consider, often falling between established boundaries for "adolescent" and "adult" age distinctions. At a time when they may be embarking on full-time paid employment or engaging in further and higher education, and making decisions that will affect their subsequent personal and career development, severely disabling chronic pain may be particularly disruptive. Yet it is not clear how common severely disabling chronic pain is in this age group.

Several previous population-based studies of chronic pain have included young adults but not provided separate estimates for this age group [10-14]. Those that have, estimate the prevalence of chronic pain in young adults as lower than older age groups, at between 4 and 14% (Table 1). In the United Kingdom, estimates of the prevalence of severely disabling chronic pain have been provided by the Grampian region study which found that 6.3% of the adult population reported severely disabling chronic pain (approximately one in every eight persons with chronic pain) [8,23]. The prevalence was strongly age-related (3.4% for 25–34 years compared with 10.6% for ≥ 75 years) and higher for women than men (9% and 7% respectively). Young adults under the age of 25 years, however, were not included.

**Table 1:** Population-based surveys of the prevalence of chronic pain in young adults

**Country/ region, year of study, reference**	**Sample frame, type of data collection**	**Method of data collection**	**Age**	**Number studied**	**Response rate**	**Definition of chronic pain (CP) and disabling chronic pain (DCP)**		**Prevalence in young adults**
Canada, Burlington (Crook et al., 1984) [15]	Random sample of households from registers from four family practices	Telephone interview (proxy report for household members)	18–91	372 h/holds (827 people)	64.4% of h/holds	CP DCP	Are you (or any member of your family over 18 years of age) *often *troubled with pain? CP + Work limitation CP + Days kept from usual activities CP + Days in bed because of pain CP + Physical function (MHI*) CP + Social function (MHI^a^)	18–30 yrs = 7.6% Not reported Not reported Not reported Not reported Not reported

UK, national, 1990 (Bowsher et al., 1991) [16]	Telephone directories	Telephone interview (proxy report for household members)	15+	1037 h/holds (2942 people)	Not reported	CP DCP	Pain lasting on or off for more than the last 3 months Unable to work or lead a normal life because of pain	15–24 yrs = 4% 15–24 yrs = 2.5%

UK, Grampian (Smith et al., 1996) [17]	Patients on repeat prescriptions for pain management registered at 2 general practices	Postal questionnaire	15+	10 712	75.5% (adj.)	CP DCP	Electronic record of repeat prescriptions for analgesia Not gathered	15–24 yrs = 4.2% -

Israel, Yeruham, 1997 (Buskila et al., 2000) [18]	Age-stratified random sample from health insurance registers	Personal interview	18–86	2210	95.2%	CP DCP	Current pain that had been present for at least 3 months (regional or widespread) CP + Lost work days in last 6 months	18–30 yrs = 9% (F); 4% (M) Not reported

Australia, New South Wales, 1997 (Blyth et al., 2001) [19]	Randomly generated telephone numbers + random sampling of one resident per household	Telephone interview	16+	17 543	70.8%	CP DCP	Pain experienced every day for 3 months in the preceding 6 months CP + Some interference with daily activities (five-point adjectival scale from 'none' to 'extreme')	15–19 yrs = 12%^E ^(F); 8%^E ^(M) 20–24 yrs = 14%^E ^(F); 12%^E^(M) 15–19 yrs = 8%^E ^(F); 7%^E ^(M) 20–24 yrs = 12%^E ^(F); 9%^E^(M) E = estimate from graph

Spain, national, 1998 (Catala et al., 2002) [20]	Electoral census; Telephone directories + quota sampling of one resident per household	Telephone interview	18–95	5000	41.7%	CP DCP	Pain during last day or week lasting more than 3 months CP + Usual activities limited by pain	18–29 yrs = 5%^E ^(F); 5%^E ^(M) 18–29 yrs = <1%

Denmark, National, 2000 (Eriksen et al., 2003) [21]	Random national sample from Danish Central Personal Register	Personal interview and postal question-aire	16+	10 066	60%	CP DCP	Chronic/longlasting pain lasting 6 months or more (excl previous/current cancer) CP + Long-lasting activity restriction CP + Quit job because of ill health CP + Absence due to illness CP + SF-36	16–24 yrs = 9% Not reported Not reported Not reported Not reported

UK, Germany, Italy, Portugal, Spain, Multinational, 1994–9 (Ohayon et al., 2003) [22]	Not reported	Telephone interview	15–100	18 980	80.4%	CP/DCP	Pain lasting 6+ months; consulted a health specialist; were taking analgesia; or pain interfered with function	<25 yrs = 13.5% (F); 9.4% (M)

The current study aimed to determine the prevalence of recent pain (within the last six months), chronic pain, and severely disabling chronic pain in young adults (aged 18–25 years) in a general population sample using established methods.

## Methods

### Sampling frame

In June 2002, a population-based postal survey of all young adults aged 18–25 years registered at 3 general practices in North Staffordshire was conducted (n = 2,389). The local population covers highly deprived wards both in terms of health deprivation and employment, skills and training deprivation [24], a finding that was confirmed by the Office of the Deputy Prime Minister, who rated Stoke-on-Trent as the 17th most deprived area of the country in 2004.

In the 2001 census, Stoke-on-Trent had a higher proportion of young adults aged 20–29 years (5.4%) compared with England and Wales (4.9%). This population was also more likely to have a long-term illness, rate their health as "not good", and have no qualifications when compared to the entire UK population.

In the United Kingdom, 98% of the population are registered with a general practice and so this provides a convenient and relatively comprehensive sample frame [25]. The accuracy of general practice registers has not been routinely audited, however previous studies have demonstrated a true address error rate ranging from 11–26% [26,27]. No information is available on the accuracy of the practices participating in this study.

Participating practices were members of North Staffordshire General Practice Research Network (GPRN) and North Staffordshire NHS Primary Care Research Consortium and as such were fully computerised, subject to annual audit, and had a commitment to, and funding for, research. Practices were selected on the basis of list size, willingness to host the study, and the absence of other research studies currently being conducted on young adults registered with the practice. Network practices are no different to other Stoke-on-Trent practices when compared using standard measures.

### Pre-pilot study

Prior to the main study, a small pre-pilot was conducted to check the ease of completion of the questionnaire and to determine the acceptability of the questions to potential participants. Although the questionnaire was 16 pages long, the participants did not find this prohibitive and did not think this would affect them responding. As a result of the pre-pilot, questions about level of education and smoking status were removed.

### Study procedures

Immediately prior to mailout the list of all potential participants was checked to exclude recent deaths or departures. General practitioners at the practices then screened the list and excluded those whom they felt were inappropriate for inclusion in a postal survey e.g. severe psychiatric illness, learning difficulties.

A standard three-stage mailout procedure was used with non-responders being sent a reminder postcard after 2 weeks and a repeat mailout of the postal questionnaire 2 weeks after this. This procedure had been used in several previous population-based postal surveys of pain in older adults in North Staffordshire with high response rates. Eligible participants were sent a letter on practice-headed notepaper from their general practitioner outlining the purpose of the study, enclosing an information sheet, and inviting them to participate. Accompanying this was a 16-page questionnaire in which participants were asked about pain experience within the last 6 months, general health and prior pain and illness.

Return of the questionnaires was taken to indicate consent to participation. On a separate page of the questionnaire respondents were asked to indicate if they gave consent to medical record review and further contact. Participants were provided with a contact telephone number at the Centre in case they wished to discuss any aspect of the study with the team of investigators.

The study was approved by North Staffordshire Local Research Ethics Committee.

### Data collection

#### Recent pain

This was assessed by a single questionnaire item adapted from a previous study of low-back pain [28]: 'Have you had any aches or pain that have lasted for a day or longer over the past six months?' (yes/no)

#### Pain location

Those reporting pain at any site within the past six months were invited to indicate the location of their pain on a manikin. 2 views were presented (front and back). The reliability of scoring has previously been demonstrated [29].

#### Pain chronicity

Participants were then asked about the duration of their most troublesome pain: 'Thinking back over the past six months, on approximately how many days have you had the most troublesome pain, which you have shaded on the manikin?' (Less than 7 days / 1 to 4 weeks / 1 to 3 months / Over 3 months). Chronic pain was defined as pain lasting for over three months in the previous six months. This corresponds to the definition of 'most days in the previous six months' proposed to capture both recurrent and continuous chronic pain [2].

#### Chronic pain severity

All respondents reporting pain over the past six months were invited to complete the Chronic Pain Grade [3] in relation to their most troublesome pain. The Chronic Pain Grade is a multi-dimensional tool, measuring persistence, intensity and duration of painful conditions. It consists of seven items that are used to classify respondents into one of four different categories. Grade I represents "low disability and low intensity pain", Grade II is "low disability-high intensity", Grade III represents "high disability-moderate intensity" and Grade IV reflects "high disability-severely limiting" pain. The validity and reliability of this measure for use in postal surveys has been investigated in a British sample that included young adults [30]. Severely disabling chronic pain was defined as chronic pain of Grade IV. This was consistent with the approach used in the Grampian region study [8].

#### Self-rated health

a single item from the SF-36 [31] was used: 'In general, would you say your health is excellent/very good/good/fair/poor?' Responses were dichotomised for analysis (excellent/very good/good versus fair/poor).

#### Anxiety/depression

the Hospital Anxiety and Depression scale (HAD) [32] was used to assess the presence of anxiety (0–21) and depression (0–21). Respondents were categorised as non-cases (0–7), possible cases (8–11), and probable cases (12–21) for both anxiety and depression using established cut-offs.

#### Further questions

the questionnaire also included further questions concerned with family and childhood experiences of pain. This information was not included in the analysis for this paper.

### Statistical analysis

The prevalence of recent pain, chronic pain, and severely disabling chronic pain were summarised as percentages of respondents with 95% confidence intervals. Levels of anxiety, depression and self-rated health were described for respondents with severely disabling chronic pain and contrasted with respondents reporting no pain.

### Investigation of non-response bias

Ethical approval for further contact with non-responders, either by telephone or post, had not been sought. We could not therefore directly ascertain whether the proportion of non-responders with severely disabling chronic pain was significantly different to that seen in responders. Instead wave analysis was used to explore the potential for non-response bias and a cross-check of all potential participants against the electoral roll was performed to explore the contribution of 'ghosts' (i.e. individuals not at the address registered with the practice) to the low response rate.

#### Wave analysis

Wave analysis is based on the assumption that non-responders may more closely resemble late responders than those who respond early. In the current study, we compared the prevalence of recent pain, chronic pain, and severely disabling chronic pain for 'early responders' (those who replied to the initial postal questionnaire within the first two weeks), 'intermediate responders' (replied within two weeks of receiving the reminder postcard), and 'late responders' (replied after repeat questionnaire mailing).

#### Electoral roll check

The names and addresses from the practice registers of all potential participants included in the survey were checked against the electoral roll for North Staffordshire. This document is held by the local council, updated annually, and includes all adults aged 18 years or over registered to vote. From 2002 registered voters were allowed to opt-out of having their details made available for commercial purposes, resulting in two editions of the electoral roll – an unedited version with restricted access, and an edited version that is commercially available. A research assistant who was blind to the response status of participants received permission from the local council to cross-check in person the practice registers against the 2002 unedited electoral roll held at the civic offices. No details from the electoral roll were removed either in hard copy or electronically. As per Pope & Croft [33] potential participants were categorised as 'ghosts' if (a) the electoral roll recorded a different surname at the same address as the practice roll, or (b) there was no entry on the electoral roll for the name and address recorded in the practice register.

## Results

13 individuals were excluded prior to mailing due to deaths and departures. 2,389 individuals were included in the survey, of whom 858 responded (crude response rate = 35.9%). 72 potential participants were excluded during the course of the survey because of deaths and departures (n = 11), ineligibility (n = 9), and responses during mailout that indicated that the intended respondent was not known at the address (n = 50) or was unable to complete the questionnaire due to illness (n = 2). The adjusted response rate was 37.0%. Response rates were significantly higher amongst women than men (difference in adjusted response rate = 15.6%; 95%CI: 11.7%, 19.5%). There was no significant difference in response rate by participating practice.

A total of 559 subjects reported suffering with any pain over the preceding 6 months, giving a 6 month period prevalence of 66.9% (allowing for 23 cases of missing data) (Table 2). The period prevalence of any pain was similar for men and women (66.6% cf 67.2% respectively). Low back pain was the single most common site reported (51.1% of respondents with recent pain).

**Table 2:** Prevalence of recent pain, chronic pain and severity of chronic pain in 835 adults

	N	Prevalence (%)	95% CI
Pain in last six months	559	66.9	63.7, 70.1
			
Duration of most troublesome pain in last six months:			
< 7 days	198	23.7	21.0, 26.7
1–4 weeks	176	21.1	18.4, 24.0
1–3 months	61	7.3	5.7, 9.3
> 3 months	119	14.3	12.0, 16.8
			
Severity of chronic pain:			
Grade I	14	1.7	1.0, 2.8
Grade II	48	5.7	4.4, 7.5
Grade III	30	3.6	2.5, 5.1
Grade IV	25	3.0	2.0, 4.4

119 respondents reported chronic pain giving a prevalence of 14.3%. Of those with chronic pain, 14 (11.8%) were classed as Grade I ("low disability and low intensity pain"), 48 (40.3%) as Grade II ("low disability-high intensity"), 30 (25.2%) as Grade III ("high disability-moderate intensity"), and 25 (21.0%) as Grade IV ("high disability-severely limiting"). The prevalence of severely disabling chronic pain in this sample was 3.0%.

An association was observed between increasing Chronic Pain Grade score and increased scores for anxiety (chi-square (8) = 21.1, p = 0.007) and depression (chi-square (8) = 14.2, p = 0.076), although only the anxiety score is statistically significant. A similar trend was noted for self rated health, with those having higher Chronic Pain Grade scores reporting poorer self-rated health (chi-square (16) = 46.8, p < 0.0001).

Compared with respondents who had no pain, those with severely disabling chronic pain were more likely to report fair or poor self-rated health, and to be classed as probable cases of anxiety and depression (Table 3).

**Table 3:** Self-reported health and anxiety and depression scores for no pain and for chronic pain (>3 months) by Chronic Pain Grade Score

	No pain No. (%)	Non chronic pain	CPG I No. (%)	CPG II No. (%)	CPG III No. (%)	CPG IV No. (%)
Self rated health						
Excellent/very good/good	258 (96)	377 (87)	12 (86)	45 (93.8)	14 (48.3)	10 (40)
Fair/poor	10 (4)	54 (13)	2 (14)	3 (6.2)	15 (51.7)	15 (60)
						
HAD Depression						
None (0–7)	196 (72)	360 (84)	10 (77)	39 (82)	17 (63)	11 (44)
Possible (8–11)	58 (21)	44 (10)	2 (15)	6 (12)	9 (33.3)	10 (40)
Probable (12–21)	20 (7)	26 (6)	1 (8)	3 (6)	1 (3.7)	4 (16)
						
HAD Anxiety						
None (0–7)	254 (93)	207 (48)	6 (46)	29 (60)	5 (18)	6 (24)
Possible (8–11)	14 (5)	109 (25)	5 (39)	9 (19)	10 (36)	6 (24)
Probable (12–21)	6 (2)	114 (27)	2 (15)	10 (21)	13 (46)	13 (52)

Of the responders with chronic pain, over 85% reported pain at three or more sites. The commonest pain sites experienced by this age group were low back pain (76.5%), neck pain (49.6%) and headaches (49.6%).

### Non-response bias

#### Wave analysis

There were no statistical differences in the reporting of either chronic pain (percentage difference: -0.7%, 95% confidence interval: -5.5%, 4.0%) or severely disabling chronic pain (percentage difference: 0.7%, 95% confidence interval: -1.7%, 3.2%) when comparing early to intermediate/late responders. (Table 4).

**Table 4:** Prevalence of recent pain, chronic pain, and severely disabling chronic pain between responders

	Recent pain		Chronic pain		Severely disabling chronic pain	
	n	%	n	%	n	%

Early responders (n = 419)	291	69.5	58	13.8	14	24.1
						
Intermediate responders (n = 137)	85	63.4	18	13.4	1	5.6
						
Late responders (n = 282)	178	63.1	43	15.2	10	23.3

#### Electoral roll check

20.1% of non-responders did not have a matching entry in the electoral roll. However, we cannot assume that all of these did not receive a survey questionnaire as 10.7% of responders also did not have a matching entry (Table 5). While it seems that 'ghosts' may have a contribution to non-response, it is not substantial in the present study.

**Table 5:** Comparison of electoral register entries for exclusions, non-responders, and responders

	Exclusions during mailing (n = 72)						Non-responders (n = 1459)		Responders (n = 858)	
	Address (n = 50)		D&D/Died/Ill (n = 13)		Ineligible (n = 9)					

	No.	%	No.	%	No.	%	No.	%	No.	%

Match										
Full name on electoral register at mailing address	11	22.0	8	61.5	2	22.2	882	60.5	613	71.4
Same surname, different first name on electoral register	4	8.0	2	15.4	5	55.6	284	19.5	153	17.8
										
Non-match										
Different surname on electoral register at mailing address	22	44.0	1	7.7	2	22.2	196	13.4	51	5.9
House not on electoral register	6	12.0	2	15.4			83	5.7	32	3.7
House outside electoral district	7	14.0					14	1.0	9	1.0
										
Total matched	15	30.0	10	76.9	7	77.8	1166	79.9	766	89.3
Total non-matched	35	60.0	3	23.1	2	22.2	293	20.1	92	10.7

## Discussion

The proportion of responders with chronic pain in this survey was 14.3%, with severely disabling chronic pain affecting 3.0% of this study population.

Comparison with previous studies suggests our estimate of the proportion of responders with chronic pain is consistent with, although at the upper end of, previous estimates. Previous reviews have failed to identify a clear explanation for differences between studies in prevalence estimates for chronic pain in the adult population [1] and similarly in young adults differences in case definition, specific age banding used to define young adults, sampling frame, and method of data collection do not appear to be consistently associated with variation in prevalence estimates between previous studies. There is, however, consensus that chronic pain is less prevalent in young adults than older age groups.

The unique contribution of this study is to provide for the first time an estimate for the prevalence of severely disabling chronic pain in young adults. This affects 3.0% of 18–25 year olds in this population and is consistent with an extrapolation of the age-related trend reported by the Grampian region study using the same measure of severity [8]. It is also plausible when compared with estimates of chronic pain in young adults using a less stringent definition of interference with daily activities from a recent Australian study [19].

This study was set in North Staffordshire, an area of the West Midlands with higher levels of deprivation and unemployment than average for the United Kingdom. Census data also informs that a higher proportion of Stoke-on-Trent residents rate their health as being 'not good' compared with rest of the population. This may have impacted on the results of this study, potentially affecting both the response rate and the pain prevalence rates, although the former is consistent with other population based estimates.

Severely disabling chronic pain in young adults appears to share some characteristics with this phenomenon in older age groups. As the Chronic Pain Grade Score increases, so do the scores for anxiety and depression (with the chi-squared analysis demonstrating statistical significance for anxiety although not for depression), with self-health more likely to be reported as being fair or poor. This is even more evident when comparing those with severely disabling chronic pain with respondents who did not report any pain. Although based on small numbers this suggests that a similar syndrome as found in older age groups may exist in young adults. It seems likely that, as in older age groups, severely disabling chronic pain in young adults is associated with a range of negative health consequences for individuals and high health care need [34].

A limitation of the present study was the low response rate. Although bias is possible even in studies with high response rates, there is greater potential for important error when response rates are low. Previous chronic pain surveys in the adult population seldom report response rates for separate age strata and so it is difficult to determine the extent of non-response in young adults in previous surveys of the adult population as a whole. According to Brattberg *et al *[10] non-response "for different sexes and age groups differed very little" (p217) whilst Mäntyselkä *et al *[14] state that "the prevalence of pain increased with age, which might be one reason why younger individuals' interest in responding was not as high as that of older individuals" (p2441). A small number of previous studies have sought to collect data on the prevalence of chronic pain from non-responders. Brattberg *et al *[10] contacted a random subsample of postal survey non-responders by telephone although the prevalence of chronic pain in this group was not separately reported. Croft *et al *[11] found the prevalence of chronic pain to be slightly lower in postal survey non-responders.

Wave analysis in the present study revealed that late responders were virtually identical to early responders regarding the proportion with recent pain or severely disabling chronic pain. Cross-checking the accuracy of the practice registers against the electoral roll suggested that 'ghosts' may have partially contributed to non-response but certainly not to the extent reported in previous studies in the adult general population, where over half of non-respondents could not be matched to the electoral roll [33]. Nevertheless, students may be registered on the electoral roll at both their term-time and vacation (typically the family home) addresses in which case the electoral roll check would under-estimate the number of young adults who did not actually receive the survey questionnaire. Out of 50 intended respondents whom we were notified were no longer at the address, their family informed us of 11 who had moved away, in some cases up to 20 years ago. Presumably others were similarly no longer at the mailing address but in the absence of notification were counted as non-respondents.

The limited insight into non-response bias gained by both wave analysis and electoral roll checks in this population argues in favour of emphasising strategies to maximise response in the design of population-based studies of younger adults. The use of survey methods developed in older age groups may not be sufficient. A number of effective strategies to increase response rates to postal questionnaires have recently been reviewed [35]. They cover the use of incentives, questionnaire length, origin, content, and appearance, and aspects of the delivery, contact, and communication surrounding the conduct of the survey itself. These should be considered in the design of future epidemiological surveys of pain in young adults, who are a difficult group for healthcare researchers to access. For future research, more innovative techniques may be required, including the increased use of technologies such as email or text messaging, and increased user involvement in questionnaire and survey design. Given the high mobility of this group (including moving out of the family home, attending university), postal surveys may not be the most satisfactory approach, although other techniques are not yet proven to be effective.

## Conclusion

Pain is a common experience in this age group, affecting 66.9% of the sample. Previously observed age-related trends in severely disabling chronic pain in older adults extend to younger adults. Although a small minority of younger adults are affected, they are likely to represent a group with particularly high health care needs. High non-response in the present study means that these estimates should be interpreted cautiously although there was no evidence of non-response bias.

## Competing interests

The author(s) declare they have no competing interests

## Authors' contributions

CM, GP and ET participated in designing the study, performing the statistical analysis and drafting the manuscript. The original study idea was conceived by PC, who also participated in the design of the study and drafting the manuscript. All authors read and approved the final manuscript.

## Pre-publication history

The pre-publication history for this paper can be accessed here:


